# Loss of Sexual Reproduction and Dwarfing in a Small Metazoan

**DOI:** 10.1371/journal.pone.0012854

**Published:** 2010-09-20

**Authors:** Claus-Peter Stelzer, Johanna Schmidt, Anneliese Wiedlroither, Simone Riss

**Affiliations:** Institute for Limnology, Austrian Academy of Sciences, Mondsee, Austria; American Museum of Natural History, United States of America

## Abstract

**Background:**

Asexuality has major theoretical advantages over sexual reproduction, yet newly formed asexual lineages rarely endure. The success, or failure, of such lineages is affected by their mechanism of origin, because it determines their initial genetic makeup and variability. Most previously described mechanisms imply that asexual lineages are randomly frozen subsamples of a sexual population.

**Methodology/Principal Findings:**

We found that transitions to obligate parthenogenesis (OP) in the rotifer *Brachionus calyciflorus*, a small freshwater invertebrate which normally reproduces by cyclical parthenogenesis, were controlled by a simple Mendelian inheritance. Pedigree analysis suggested that obligate parthenogens were homozygous for a recessive allele, which caused inability to respond to the chemical signals that normally induce sexual reproduction in this species. Alternative mechanisms, such as ploidy changes, could be ruled out on the basis of flow cytometric measurements and genetic marker analysis. Interestingly, obligate parthenogens were also dwarfs (approximately 50% smaller than cyclical parthenogens), indicating pleiotropy or linkage with genes that strongly affect body size. We found no adverse effects of OP on survival or fecundity.

**Conclusions/Significance:**

This mechanism of inheritance implies that genes causing OP may evolve within sexual populations and remain undetected in the heterozygous state long before they get frequent enough to actually cause a transition to asexual reproduction. In this process, genetic variation at other loci might become linked to OP genes, leading to non-random associations between asexuality and other phenotypic traits.

## Introduction

The ubiquity of sexual reproduction is an evolutionary puzzle because asexuality should have major theoretical advantages [1], [2]. Thus many hypotheses have been formulated to explain the success of the sexual reproductive mode, or why asexual lineages rarely persist over the longer term [3], [4], [5], [6]. The mechanism of origin of such lineages has important implications on their evolutionary success, or failure, because it affects their initial genetic makeup and variability [2], [7]. The origin of new asexual lineages is typically viewed as a process that randomly freezes small proportions of the genetic variation of a sexual population [8], [9]. This likely applies to the common modes of origin: hybridization, polyploidization, or infectious origin [7]. Likewise, dominant sex-limited Meiosis-suppressors [10], [11] should result in asexual lineages that are a random sample of the genotypes of sexual populations. By contrast, gradual transitions towards asexuality would allow for hitchhiking effects, such that other traits may become linked to parthenogenesis. Such gradual transitions to asexuality would be a plausible scenario if the propensity to reproduce (a)sexually was determined by additive genetic variation, or if asexuality was caused by a single non-dominant allele. To date, information on such mechanisms is very scarce. We are only aware of one study, which showed a recessive mutation to underlie a transition from parthenogenetic production of males (arrhenotoky) to parthenogenetic production of females (thelytoky) in workers of the cape honey bee [12]. This is not directly equivalent to a transition to obligate parthenogenesis, since the queens are still capable of sexual reproduction.

We investigated transitions to obligate parthenogenesis in the monogonont rotifer *Brachionus calyciflorus*. Monogononts normally reproduce by cyclical parthenogenesis, an alternation between ameiotic parthenogenesis and sporadic sexual episodes [13]. But there are also several documented cases on *Brachionus* strains that have permanently lost the ability to reproduce sexually [14], [15], [16], [17], [18]. In cyclical parthenogenetic rotifers, sex is initiated with the production of sexual females, whose oocytes undergo meiosis and develop into haploid males (if not fertilized), or diploid diapausing eggs (if fertilized). In *Brachious* and several other Monogonont rotifers, the production of sexual females is induced at high population densities by a chemical that is produced by the rotifers themselves [19], [20], [21], a process analogous to *quorum sensing* in bacteria [22]. Obligate parthenogens are not able to produce sexual females, thus they also lack males and diapausing eggs. It has been demonstrated that this inability is caused by a loss of responsiveness to the chemical signal that induces sex [18]. As a consequence, populations of obligate parthenogens can grow to extremely high population densities, without ever inducing sex, whereas cyclical parthenogens readily induce sexual reproduction as soon as population densities exceed one female per ml [23]. We used this difference to screen for obligate parthenogens in our experimental *Brachionus* clones.

Two experimental procedures were used to determine the inheritance pattern of obligate parthenogenesis: self-fertilization and cross-fertilization. Clones in *B. calyciflorus* are made up of all asexually produced descendants of a single female, including sexual females and males. Since each male represents a random haploid sample of the female genome, mating between males and sexual females of the same clone is genetically equivalent to selfing. Alternatively, clones of *B. calyciflorus* can be cross-fertilized, if virgin sexual females of one clone are presented to males of another clone.

## Results

### Mendelian inheritance of obligate parthenogenesis

**Figure 1:**
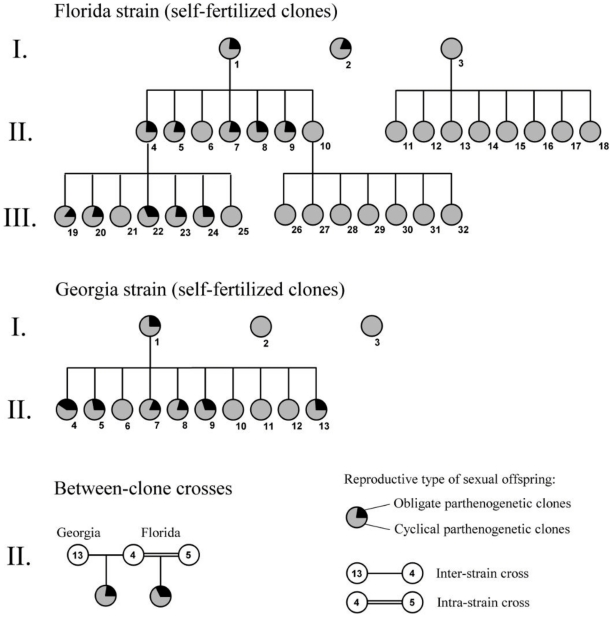
Mendelian inheritance of obligate parthenogenesis in two strains of the rotifer *Brachionus calyciflorus*. The figure shows an overview of all experimental clones, represented by numbered pie charts, which were propagated either by self-fertilization or experimental crosses. Roman numbers indicate successive sexual generations; Arabic numbers indicate individual clones of the two rotifer strains. Pie charts display the proportion of obligate *vs*. cyclical parthenogens among the sexually produced offspring clones of each clone. We analyzed 45 clones and determined the reproductive type in 88±5.2 (s.e.m.) of their sexual offspring clones, giving a grand total of 3962 analyzed clones.

During the initial phase of our study, several clones were established from two geographic strains of *Brachionus calyciflorus* (a Georgia and a Florida strain; see Materials and Methods). After self-fertilizing these clones, we discovered that some produced diapausing eggs whose hatchlings turned out to be obligate parthenogenetic clones, at a frequency of approximately 25% (the remaining 75% hatchlings were cyclical parthenogenetic clones; see Fig. 1: Florida clones 1 and 2, and Georgia clone 1). Other clones produced only cyclical parthenogenetic clones upon self-fertilization (see Fig. 1: Florida clone 3, and Georgia clones 2 and 3). These percentages suggested that obligate parthenogens could be homozygous for a recessive allele, *op* (for *o*bligate *p*arthenogenesis) causing a permanent loss of sexual reproduction: Heterozygotes, which still contain one wild-type allele (*+/op* clones), would be phenotypically cyclical parthenogens, but produce obligate parthenogenetic clones (*op*/*op*) upon selfing at a 3∶1 ratio (CP∶OP), whereas homozygotes for the wild-type allele (*+/+*) are also cyclical parthenogens, but should produce cyclical parthenogens only.

To probe further into this potential mechanism, subsequent generations of selfing were analyzed. Indeed, we could confirm that all clones obtained by self fertilization of *+/+* clones were cyclical parthenogens (Fig. 1: descendants of Florida clones 3 and 10). By contrast, some of the cyclical parthenogenetic clones resulting from selfing of heterozygotes (*+/op*) consistently showed the expected segregation into the two discrete phenotypes at a 3∶1 ratio, which indicates that they received the *+/op* genotype from their parental clone (Fig. 1: Florida clones 1 and 4, Georgia clone 1). The 3∶1 segregation pattern was consistently found in the Florida strain (Replicated G-Test: Twelve “*+/op* clones” analyzed, 1221 offspring clones typed, on average 23.4% OP clones, *G*_het_ = 7.6, *df* = 11, *P* = 0.748, *G*_pooled_ = 1.64, P = 0.2) and in the Georgia strain (Seven “*+/op* clones” analyzed, 548 offspring clones typed, on average 27.5% OP clones, *G*_het_ = 9.9, *df* = 6, *P* = 0.126, *G*_pooled_ = 1.86, *P* = 0.17). The coherence of our experimental procedure for self-fertilization with genetic selfing was independently confirmed by genotyping the experimental clones with AFLP markers and showing an increase of homozygosity of such markers in the selfed lines (Appendix S1)

Several additional lines of evidence support our conclusions about the Mendelian inheritance. First, cross-mating between two heterozygous clones of the Florida strain resulted in 31% obligatory parthenogenetic clones (Fig. 1, clones 4 and 5 of the Florida strain). This confirmed that the observed segregation patterns were not an artefact of self-fertilization. Second, a cross between two *+/op* clones of the Florida and Georgia strain resulted in 23% obligate parthenogenetic clones (Fig. 1, clones “Georgia 13” x “Florida 4”), which additionally suggests that obligate parthenogenesis in the Florida and Georgia strain might be caused by a mutation in the same gene (complementation). Third, selfing of heterozygous clones (*+/op* ) resulted in a 1∶2 segregation of homozygous clones (*+/+*) *vs.* heterozygous clones (*+/op*) in CP clones. Overall, we found that eight of 24 CP daughter clones were *+/+*, while 16 were *+/op* (see Fig. 1: Selfed offspring of the Florida clones 1 and 4, and Georgia clone 1).

The transition to OP in our clones of *Brachionus calyciflorus* was permanent: Some of our oldest obligatory parthenogenetic clones were hatched more than three years ago and have been cultured in laboratory since then (i.e., for ∼500 asexual generations). We never observed any males or other sexual stages in such cultures.

### Dwarfing in obligate parthenogens

**Figure 2:**
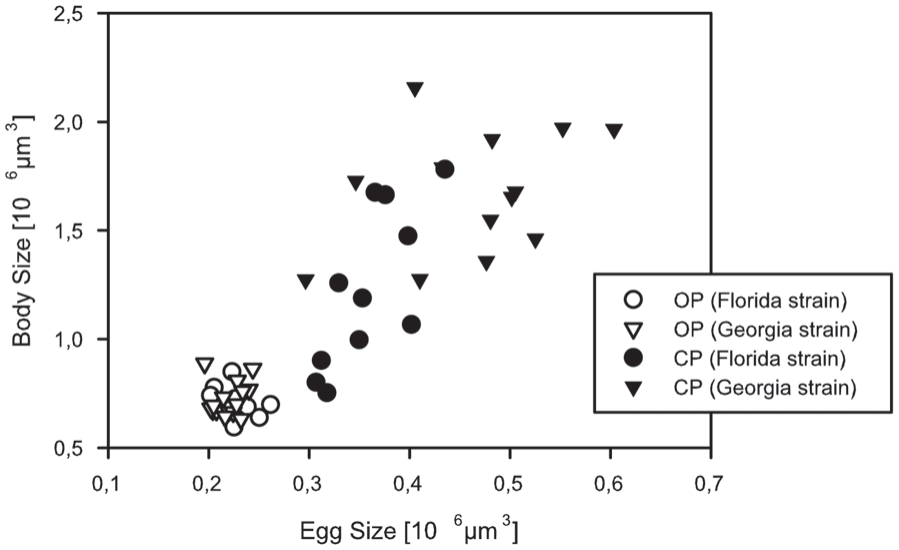
Dwarfing in obligate parthenogenogenetic clones of the rotifer *Brachionus calyciflorus*. Each symbol represents the mean value of egg size and female body size for different clone (Sample sizes for each clone: Eggs: n = 24±1.4; Females: n = 15±0.9; mean ± s.e.m.). Open symbols: obligate parthenogenetic clones (OP); closed symbols: cyclical parthenogenetic clones (CP), Circles: clones of the Florida strain; Triangles: clones of the Georgia strain. In total, 40 clones were analyzed (23 OP and 17 CP).

Interestingly, OP clones were significantly smaller in body/egg size than CPs (Welch's test for equality of means, Body size: *F*_1,24.8_ = 86.1, *P*<0.001, Egg size: *F*_1,24.9_ = 117.9, *P*<0.001). While there was considerable variation in body/egg size among CPs, the size of OPs seemed to be uniformly small (Fig. 2). In fact, the variances in these traits were significantly different between CPs and OPs (Levene's test, Body size: *W* = 34.56, *P*<0.001, *df* = 1, 45, Egg size: *W* = 34.04, *P*<0.001, *df* = 1, 45). Since OPs were on average ∼50% smaller than CPs, we initially hypothesized that obligate parthenogens might be haploid. Haploid females are very rare in the animal kingdom, but given the haplo-diploid sex determination in monogonont rotifers and the fact that the haploid males are dwarfed[13], there would have been the theoretical possibility that obligate parthenogenetic females could be “feminized males” (e.g., see[24]). However, we could clearly discard this hypothesis based on flow cytometric measurements of genome size - obligate and cyclical parthenogens had identical nuclear DNA contents (Appendix S2).

The smaller body size of obligate parthenogens did not imply lower fitness. Overall, OPs and CPs had similar mean life spans of 4.7 days ±0.17 (s.e.m.) and 4.3 days ±0.11, respectively (Students *t*-test, *t* = 1.65, *P* = 0.106, *df* = 42). In terms of fecundity, OPs even performed significantly better than CPs, having on average 13.1±0.93 (s.e.m.) *vs*. 9.3±0.95 offspring per female (Students *t*-test, *t* = 2.88, *P* = 0.006, *df* = 42). If compared to other animal groups, it may be puzzling that small body size does not automatically correlate with lower fecundity. However, *Brachionus* rotifers produce their eggs sequentially, rather than in clutches and attach them externally to the posterior end until they hatch. Hence there is no body size constraint to fecundity as in many other organisms [25].

## Discussion

Our evidence based on the analysis of selfed lines and crossing experiments unequivocally supported the hypothesis that obligate parthenogenesis in *Brachionus calyciflorus* is inherited in a Mendelian fashion. We are aware that this does not necessarily imply that OP is caused by a mutation at a single locus. For instance, epistatic interactions among several unlinked loci on the same chromosome may result in a similar inheritance pattern, as long as there is no recombination among such loci. However, given that OP is a recessive trait involving a simple loss of function ( = loss of responsiveness to the sexual signal[18]), a mutation in a single gene is probably the most parsimonious explanation. This mechanism of inheritance implies that genes causing OP may evolve within sexual populations and remain undetected in the heterozygous state long before they get frequent enough to actually cause a transition to asexual reproduction.

The association of small body size with obligate parthenogenesis is very unusual; in fact we are not aware of any reports from other animals or plants displaying such a pattern. In most cases, obligate parthenogens are of similar size, or even larger than their closest sexual relatives – the latter can usually be attributed to polyploidy [26]. Nevertheless, an earlier study has reported precisely the same association of small body size and obligate parthenogenesis in an artificially selected laboratory population of *Brachionus calyciflorus* [27]. Our observation, that even the selfed offspring of a clone may display such size differences (i.e., individuals that are genetically highly similar), suggests that the genetic predisposition for dwarfism is tightly linked to obligate parthenogenesis, possibly determined by a locus in close proximity to the OP allele, an epistatic interaction, or due to pleiotropy.

To our knowledge, the *op* allele has no obvious effects in heterozygote carriers, neither on sexual reproduction, nor on body size, survival, or fecundity. However, heterozygotes might exhibit higher density thresholds for sex induction as compared to homozygotes for the wild-type allele. Our bioassay was not designed to detect such subtle differences. Rather, we used population densities that were far above normal density thresholds for sex induction, in order to exclude the possibility of erroneously assigning OP to clones that just did not receive a sufficiently strong cue. In future studies, however, different sex induction assays could be used to test whether heterozygotes are less responsive to the chemical signal that induces sex. Theoretical studies suggest that higher sexual thresholds may be adaptive under certain environmental conditions [28], [29], and there is also empirical evidence for such variation [30].

So far, nothing is known about the frequency and distribution of the *op* allele in natural populations. Such alleles might be quite common, given that several independent studies have reported losses of sexual reproduction in *Brachionus calyciflorus* [14], [15], [16], [17], [18]. A promising avenue for future research might thus be to map the locus responsible for obligate parthenogenesis. This would enable screens for heterozygote carriers in natural populations, and potentially provide clues about the conspicuous link between obligate parthenogenesis and dwarfing.

## Methods

### Culture methods

Two strains of the rotifer *Brachionus calyciflorus*, a Florida and a Georgia strain, were used in this study (kindly provided by J.J. Gilbert, Dartmouth College, USA). The geographic origin of these two strains has been described in detail in an earlier study [31]. The rotifers were cultured in COMBO medium [32] with the unicellular algae *Chlamydomonas reinhardii* as food source (Strain: SAG11-32b, Sammlung fuer Algenkulturen, Goettingen, Germany). Algae were supplied at *ad libitum* concentrations (∼400,000 cells ml^−1^). Cultures were kept at 24°C and continuous illumination was provided with daylight fluorescent bulbs (30–40 µEinstein m^−2^ s^−1^ for rotifers; 200 µEinstein m^−2^ s^−1^ for algae). Clonal cultures of rotifers were re-inoculated twice per week by transferring 20 asexually reproducing females to fresh culture medium provided in 20 ml petri dishes.

### Selfing and crossing procedures

In addition to the clonal rotifer cultures, we established pedigrees of sexually propagated lines, either by self-fertilization or crossing of clones. Selfing is possible in monogonont rotifers, because one rotifer clone can give rise to males, which produce haploid sperm, and at the same time it can give rise to sexual females, which produce haploid oocytes [25]. Thus if one clone is grown in a mass culture, it will produce diapausing eggs, which are sexually recombined offspring of itself. Diapausing eggs were harvested from dense clonal cultures and concentrated by sedimentation. Aliquots of the concentrated suspension were distributed among several micro centrifuge tubes, dried in a rotation evaporator at 30°C, and subsequently stored in the dark at 4–7°C. This procedure kills all live individuals and asexually produced eggs, except for the drought-resistant sexual diapausing eggs. After a storage period of at least 2–3 weeks, hatching was induced by flooding the dried eggs with food suspension and incubation at 25°C and high light intensities (200 µEinstein m^−2^ s^−1^). Usually after 24 hours, the first hatchlings started to emerge and clonal cultures were initiated. Crossings between clones were accomplished by presenting freshly hatched virgin females with males of another clone. Briefly, female eggs were harvested from mass cultures at population densities of 10–100 ind. ml^−1^, i.e., when the population initiated sexual reproduction. Individual female eggs were isolated into 24-well tissue culture plates, filled with 0.5 ml food suspension, and 8 actively swimming males of the opposite clone were added to each well. After 24 hours, females were transferred to new wells with fresh food suspension. The fertilized females were cultured for another 48–72 h, until they had produced 2–3 diapausing eggs. Finally, all diapausing eggs were combined into a micro centrifuge tube filled with 0.1 ml COMBO medium, dried in a rotation evaporator, and stored in the same way as diapausing eggs obtained by self-fertilization (see above).

### Detection of reproductive mode

The reproductive mode of clones (OP or CP) was determined using experimental screens. In such screens, clonal cultures were propagated by transferring 5–6 asexual females every 3–4 days into fresh algal suspension (5 ml volume) provided in 6-well tissue culture plates. Between these transfers, the population size typically increased to about 40–60 individuals. Populations of OP clones usually reached more than hundred individuals per well after 3–4 days. After each transfer, the old culture was examined for sexual stages (females with male eggs, males, diapausing eggs). These screens were run for 3 weeks (i.e. approximately ten asexual generations). Clones that did not show any sexual stages during this time were considered as obligate parthenogens. We can be sure that this experimental duration is sufficient for establishing the reproductive mode, since on several instances we cultured clones for a much longer time (several weeks to months) – yet we never observed any sexual stages in clones that had been formerly classified as obligate parthenogens. Some of our oldest OP clones were hatched more than three years ago, yet males or other sexual stages in such cultures were never observed.

### Size measurements

For body size and egg size measurements, newborn females (age <6 h) were isolated and cultured individually at *ad libitum* food concentrations, with daily transfers to fresh food suspension. At the age of three days, females were adult and usually carried several eggs. These females were fixed in Lugol's solution and transferred to Plankton sedimentation chambers. Body size was measured using inverted microscopy at 200-fold magnification. Body volume was estimated from three distance measurements on each individual (total length, widest breadth, breadth at the anterior end) according to [33]. Egg volume was estimated from length-breadth measurements, assuming that eggs have the shape of ellipsoids of revolution. In most cases, 20 females and 30 asexual eggs per experimental clone were measured (in total: 25 CP clones and 23 OP clones).

### Survival and fecundity

Survival and fecundity of CPs *vs*. OPs were estimated from life tables of various clones of the Florida and Georgia strain. For the purpose of the present study, we analysed only the most basic parameters of the life tables: survival and fecundity. A more detailed analysis of these life tables has been submitted elsewhere, along with competition experiments between CPs and OPs, (Stelzer, submitted manuscript). Briefly, cohorts of individuals for each clone were established from hatchlings of asexual eggs (age <6 h). Twelve individuals per clone were kept in 1 ml food suspension in 24-well tissue culture plates and were transferred to fresh food suspension every 12 hours until death. Survival was checked at each transfer and fecundity was measured by collecting the offspring produced by each female within the preceding 12-h interval. We calculated R_0_, lifetime reproductive success, as the average number of offspring produced by a female, as well as mean lifespan for each clone. A total of 20 cyclical parthenogenetic clones and 24 obligate parthenogenetic clones were analyzed.

## Supporting Information

Appendix S1 Methods and results of AFLP genotyping, which was used to confirm selfing and experimental crosses.
(0.11 MB PDF)

Appendix S2 Flow-cytometric measurements of genome size of cyclical vs. obligate parthenogenetic clones.
(0.15 MB PDF)
