# Supplementary material for: Loss of Sexual Reproduction and Dwarfing in a Small Metazoan
Source: PLoS One. 2010 Sep 20;5(9):e12854. doi: 10.1371/journal.pone.0012854 (PMC2942836; doi:10.1371/journal.pone.0012854)
Supplement: Appendix S1 — Methods and results of AFLP genotyping, which was used to confirm selfing and experimental crosses. (0.11 MB PDF) [file pone.0012854.s001.pdf]

Appendix S1

Claus-Peter Stelzer, Johanna Schmidt, Anneliese Wiedlroither, and Simone Riss

"Loss of sexual reproduction and dwarfing in a small metazoan"

AFLP genotyping

The AFLP technique[1] was used to confirm successful crosses and selfing of *B. calyciflorus.* DNA was isolated from frozen biomass preserved in 70% ethanol. Clonal cultures of rotifers were grown in 1L glass bottles with *Chlamydomonas reinhardtii* as food algae and aerated with sterile air through a glass tube. The cultures were initiated with 50-100 females and were grown for 7-10 days until they reached population densities of 10-100 individuals per mL. Rotifer biomass was harvested with 30µm sieves, resuspended in sterile culture medium and starved for 2h. This procedure was repeated twice to ensure that rotifers completely emptied their guts. Cleaned rotifer biomass was resuspended in 70% ethanol. Before DNA isolation, ethanol was removed by centrifugation and washing with 1 ml of sterile H_2O. DNA was isolated using the DNeasy Blood & Tissue kit (Qiagen). DNA was eluted with 50-100 µl of pre-warmed 1X $TE_{0.1}$ buffer (20 mM Tris-HCl, 0.1 mM EDTA, pH 8.0). Concentration and purity of DNA were measured with a NanoDrop spectrophotometer (ThermoSCIENTIFIC), followed by dilution of DNA with sterile H_2O to the required concentration of 9.1 ng/µl. Restriction-ligation reaction and preselective amplification of the AFLP procedure were carried out using the AFLP® Ligation and Preselective Amplification Module for Small Plant Genomes of 50-500Mb (Applied Biosystems). Briefly, in the restriction-ligation reactions 50 ng of genomic DNA were digested and ligated to EcoRI- and MseI-adaptors in 11 µl volumes containing 1.1 µl 10X T4 DNA ligase buffer with ATP, 1 U MseI, 5 U EcoRI, 1 WeissU T4 DNA ligase, 0.577 µl 1 mg/mL BSA (all: New England Biolabs), 1.1 µl 0.5 M NaCl, and 1 µl MseI- and 1 µl EcoRI-adaptors (Applied Biosystems). Restriction-ligation reactions were carried out for 2 h at 37°C in a thermal cycler. Afterwards the DNA was diluted 18.18-fold with $TE_{0.1}$ buffer. For the preselective amplification, 15 µL AFLP Core Mix, 1 µL AFLP preselective primer pairs (both: Applied Biosystems) and 4 µL diluted DNA prepared by restriction-ligation were combined in a PCR reaction tube. PCR amplification was carried out in a thermal cycler using the following program: 72 °C for 2 min.; 20 cycles: 94 °C for 20 sec., 56 °C for 30 sec., 72°C for 2 min.; 60 °C for 30 min.. The product was diluted 20-fold with $TE_{0.1}$ buffer. For selective amplification, initially 16 different combinations of three fluorescent-labelled primers binding to the EcoRI- adaptor and eight unlabelled primers binding to the MseI-adaptor were tested to find the most suitable primers for genotyping

(primer sequences: KeyGene, synthesis: Eurofins MWG Operon). A detailed list of the primer sequences and the combinations used for the final analysis can be found below (Table S1).

Table S1: AFLP Primer combinations tested in this study. The combinations which gave the most signals and were finally chosen are marked with (+). Core sequences of the primers were EcoRI: 5'-Dye-GACTGCGTACCAATTC-NN-3' and MseI: 5'-GATGAGTCCTGAGTAA-NNN-3'. The nomenclature for AFLP primers according to KeyGene (http:\\www.keygene.com) is given in brackets.

	FAM-EcoRI-AC (E12)	TAMRA-EcoRI-TT (E26)	JOE-EcoRI-AG (E13)
MseI-CAA (M47)	+		+
MseI-CAC (M48)			
MseI-CAG (M49)			+
MseI-CAT (M50)	+		+
MseI-CTA (M59)	+		+
MseI-CTC (M60)	+		
MseI-CTG (M61)			
MseI-CTT (M62)			

For the PCR reaction 3 µl diluted product, 1 µl MseI-primer (at 5 µM), 1 µl fluorescent-labelled EcoRI-primer and 15 µl AFLP core Mix were mixed. The PCR conditions were: 94°C for 2 min.; 10 cycles: 94°C for 20 sec., 66°C – 1 °C/cycle for 30 sec., 72 °C for 2 min., 20 cycles: 94°C for 20 sec., 56°C for 30 sec., 72 °C for 2 min.; 60 °C for 30 min.). For fragment analysis, 5 µl of fluorescent-labelled product were mixed with 14.4 µl of Hi-Di™ Formamide (Applied Biosystems), 14.9 µl H_2O and 0.2 µl GeneScan™ 500 ROX™ Size Standard (Applied Biosystems), denatured and analyzed on an ABI 3730 capillary sequencer (Applied Biosystems) at the Core Facility Molecular Biology of the Medical University of Graz. Trace files were analyzed in Genemarker® (Softgenetics). We analyzed fragment sizes of 50-500 bp and only considered markers with signal intensities of >150 RFU (Relative Fluorescent Units) if signal was present, or <35 RFU, if signal was absent. AFLP Markers producing weak or ambiguous signals (i.e., between 35-150 RFU), were not considered.

AFLP markers were used to confirm self-fertilization and crossings in our experimental clones. Since AFLPs are dominant genetic markers, it is difficult to determine whether an individual is heterozygous for a particular marker. However, if a large number of markers are analyzed, self-fertilization should result in a systematic loss of alleles at formerly heterozygous loci. We searched for such losses in two subsequent generations of our selfed clones in the Florida strain. Briefly, we analyzed six clones of Generation II and five clones of Generation III (see Fig. 1 in main text). Clone "Florida 4" of Generation II was our focal genotype, since it was the one from which the clones of Generation III were established. Overall we found 93.2%, of a total of 118 marker alleles, to be identical in all 11 clones, which suggested that these clones were already homozygous for the majority of our investigated AFLP loci. This observation is consistent with a history of multiple generations of repeated selfing or inbreeding, which likely happened before our experiments (note that the Florida strain had been kept in the lab for several years). However, we also found three cases in which alleles that were present in the focal clone ("Florida 4") had apparently been lost in some of its selfed offspring. To double check whether these markers were indeed heterozygous in the focal individual, we analyzed band intensity variation in all AFLP markers, both in the focal individual, and among its sisters and its selfed offspring. For homozygous markers, we can expect uniformly low coefficients of variance (CV) of band intensities. By contrast, markers which are heterozygous in some individuals, but homozygous in others, should exhibit higher CVs due to the variation in initial DNA template prior to the AFLP reaction. To obtain an estimate for such band intensity variation, we first calculated the CVs for the band intensities of AFLP markers *that were present in all 11 individuals*, hence likely candidates for homozygous markers. On average, such variation was 18.9% (Fig. S1). We then searched for AFLP markers that were present in our focal clone but absent in any of the other clones, hence likely candidates for markers that are heterzygous in the focal clone. Again we checked the coefficient of variation in the AFLP marker signals: The variation in one marker was only slightly elevated (24.5%), yet the other two markers had CVs that were far higher than the majority the other AFLP markers tested (43.2% and 58.1%, see arrows in Fig. S1). Closer inspection of signal intensities showed that all these markers had signal intensities in the focal clone, which were approximately 50% of the highest signals for each marker. Thus they likely represented alleles heterozygous in the "Florida 4" clone (Table S2). All together, these observations confirm that our experimental clones were indeed highly homozygous as a result of repeated selfing.

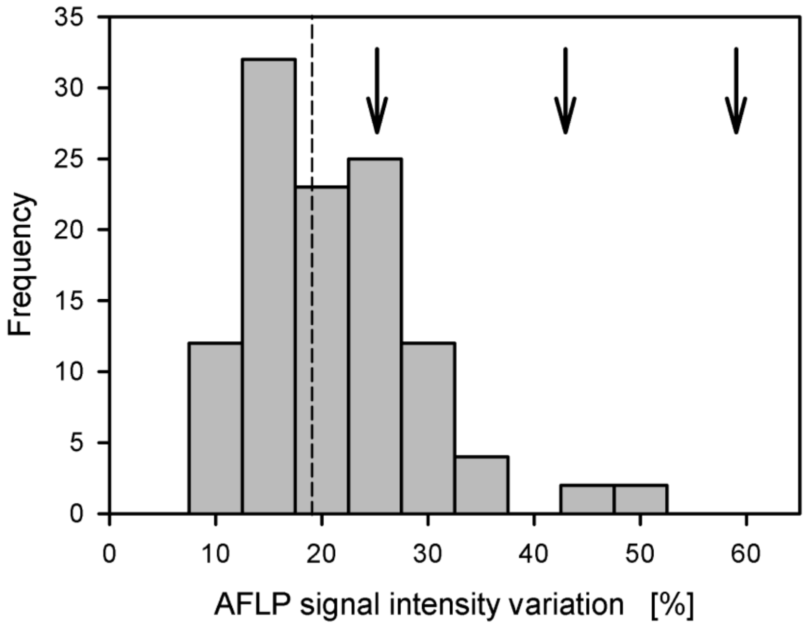

Fig. S1: Screen for AFLP markers that were lost upon selfing. Bar chart displays signal intensity variation of markers that were present in all 11 individuals studied (i.e., predominantly homozygous markers); the dashed line indicates their mean value (18.9%). Arrows indicate the coefficients of variation of three different AFLP markers, which had been apparently lost upon to selfing.

Table S2: Loss of heterozygosity upon selfing. The table shows the signal intensities in RFUs (=Relative Fluorescence Units) in 11 different clones of the Florida strain for three different AFLP markers. The focal clone, "Florida 4" (the one which was selfed), is displayed in **bold**.

Generation	Clone Designation (cf. Fig. 1)	AFLP Marker E12/M47_113	E13/M50_235	E13/M59_115
II.	Florida 10	614	224	-
II.	Florida 6	300	-	199
II.	Florida 5	376	413	216
II.	Florida 9	204	-	232
II.	*	-	822	244
II.	**Florida 4**	**340**	**260**	**165**
III.	*	291	333	221
III.	Florida 23	-	-	251
III.	Florida 21	227	155	157
III.	Florida 24	615	-	-
III.	*	-	521	345

* not used/displayed in pedigree analysis (Fig. 1)
- Allele not detectable (<35 RFU)

We also used AFLP markers to genetically confirm our experimental crosses between the Georgia and Florida strain. In the two parental clones of this crossing, “Florida 4” and “Georgia 13” (cf. Fig. 1 in main text), we identified 6 unique alleles, and analyzed the distribution of these alleles in the sexual offspring produced by crossing these two clones. We found that the offspring received alleles from the “Florida 4”-parent, as well from the “Georgia 13”-parent, which conforms to the expectation of sexual recombination between the two parental clones (see Table S3).

Table S3: Genetic confirmation of crosses between clones of *B. calyciflorus*. We tested three sexual offspring of two parental clones, Florida 4 and Georgia 13, respectively. The replicates of the parental clones resemble biological replicates, i.e. completely independent biomass preparations of a clone. Entries of the table denote presence (“1”) or absence (“0”) of an allele. AFLP markers were named according to the official nomenclature for AFLP primer combinations (http:\\www.keygene.com) with the last number giving the size of the fragment considered (in base pairs).

Clone	Replicate	AFLP markers					
		E12/M50_74	E12/M50_99	E12/M59_91	E13/M47_71	E13/M50_131	E13/M50_211
Parents:							
Florida 4	1	0	0	0	1	0	1
	2	0	0	0	1	0	1
	3	0	0	0	1	0	1
Georgia 13	1	1	1	1	0	1	0
	2	1	1	1	0	1	0
	3	1	1	1	0	1	0
	4	1	1	1	0	1	0
Offspring:							
GxF 1		1	0	1	1	1	1
GxF 2		0	0	0	1	1	1
GxF 3		1	0	0	1	1	1

References

1. Vos P, *et al.* (1995) AFLP: a new technique for DNA fingerprinting. *Nucl. Acids Res.* 23(21):4407-4414.
