# Supplementary material for: Loss of Sexual Reproduction and Dwarfing in a Small Metazoan
Source: PLoS One. 2010 Sep 20;5(9):e12854. doi: 10.1371/journal.pone.0012854 (PMC2942836; doi:10.1371/journal.pone.0012854)
Supplement: Appendix S2 — Flow-cytometric measurements of genome size of cyclical vs. obligate parthenogenetic clones. (0.15 MB PDF) [file pone.0012854.s002.pdf]

Appendix S2

Claus-Peter Stelzer, Johanna Schmidt, Anneliese Wiedlroither, and Simone Riss

"Loss of sexual reproduction and dwarfing in a small metazoan"

Genome size measurements

The hypothesis that obligate parthenogens are haploid was tested by measuring the genome sizes of two experimental clones (CP: the Florida clones 4 and 5, see Fig. 1; OP: two sister clones of the same generation, and also deriving by self-fertilization from the parental clone "Florida 1"). Genome size in obligate and cyclical parthenogenetic clones of the Florida strain was measured using flow cytometry. For these measurements, clonal cultures of rotifers were grown in 1L glass bottles with *Chlamydomonas reinhardtii* as food algae and aerated with sterile air through a glass tube. The cultures were initiated with 50-100 females and were grown for 7-10 days until they reached population densities of 10-100 individuals per mL. Rotifer biomass was harvested with 30µm sieves, resuspended in sterile culture medium and starved for 2h. This procedure was repeated twice to ensure that rotifers completely emptied their guts. Cleaned rotifer biomass was resuspended in 10 mL Stock solution (3.4mM Trisodium citrate dihydrate, Nonidet P40 at 0.1% v/v, 1.5 mM Sperminetetrahydrochloride, 0.5mM Trishydroxymethyl-aminomethane, pH 7.6), concentrated by centrifugation (1min at 1000g) and buffer was removed to 0.3-1 mL (depending on the amount of initial biomass). This procedure typically resulted in harvests of 10-20µl concentrated biomass. Rotifer biomass was ground on ice with 50 strokes in a 1mL Dounce tissue homogenizer, to free individual nuclei. Large debris was removed by filtration through a 35µm mesh nylon sieve. A detergent-trypsin method[1] was used to prepare the nuclei for flow-cytometric analysis. Briefly, 100µl of the homogenized cell suspension was digested by addition of 450µl of 0.003% Trypsin for 10 min at room temperature. To prevent further degradation, 0.05% trypsin inhibitor was added (this solution also included 0.01% RNAse A) and the samples were incubated for another 10 min. Finally, samples were stained with propidium iodide at a concentration of 50µg/mL. Stained samples were kept overnight on ice in the dark. Flow cytometric analysis was performed in a FacsCalibur flow cytometer (BD Biosciences) using the manufacturer's instructions. As internal standard of known genome size we used the fruit fly, *Drosophila melanogaster* (strain ISO-1, nuclear DNA content: 0.35pg[2]). Ten female *Drosophila* heads were homogenized in 0.5 ml Stock solution with 15 strokes in the Dounce tissue homogenizer, and 100µl of this homogenate was co-prepared with the rotifer samples and stained in exactly the same way. Rotifer samples and *Drosophila* standard were first run

separately on the cytometer, to identify the position of the peaks, and then combined into the same sample and measured again. Each sample was analyzed until a pre-specified number of 7,000 events (i.e., particles registered by the fluorescence detectors) were reached, typically at a rate ~30 events per second. When rotifer samples were measured in combination with the internal *Drosophila* standard, the number of counted events was increased to 15,000. Three replicates (i.e., independent biomass preparations) were used for each of the four rotifer clones. The flow cytometry measurements confirmed that both reproductive types, cyclical parthenogens and obligate parthenogens, had the same ploidy (Fig. S2, S3). The mean nuclear DNA contents of the four clones were 0.262, 0.261, 0.254 and 0.262 pg, and they were not significantly different from each other (Fig. S2, ANOVA, $F_{3,11}$=0.716, P=0.57). Since we used mass cultures for these analyses, we often observed an additional peak in CPs, which was exactly half of the diploid peak (Fig. S3). This peak was caused by haploid males, which were usually also present in CP mass cultures. We never observed a "male peak" in obligate parthenogens.

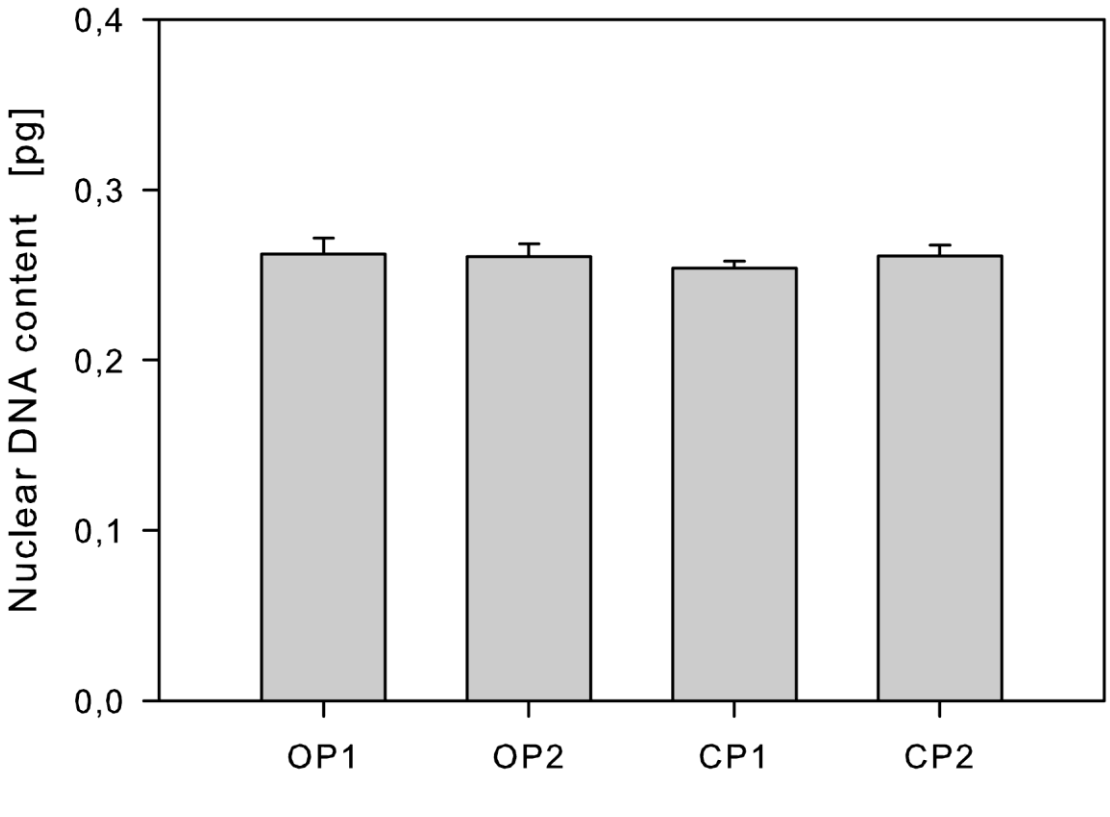

Fig. S2: Flow-cytometric measurements of genome size in two obligate parthenogenetic clones (OP1 and OP2) and two cyclical parthenogenetic clones (CP1 and CP2). Means and standard deviations (n=3, per clone).

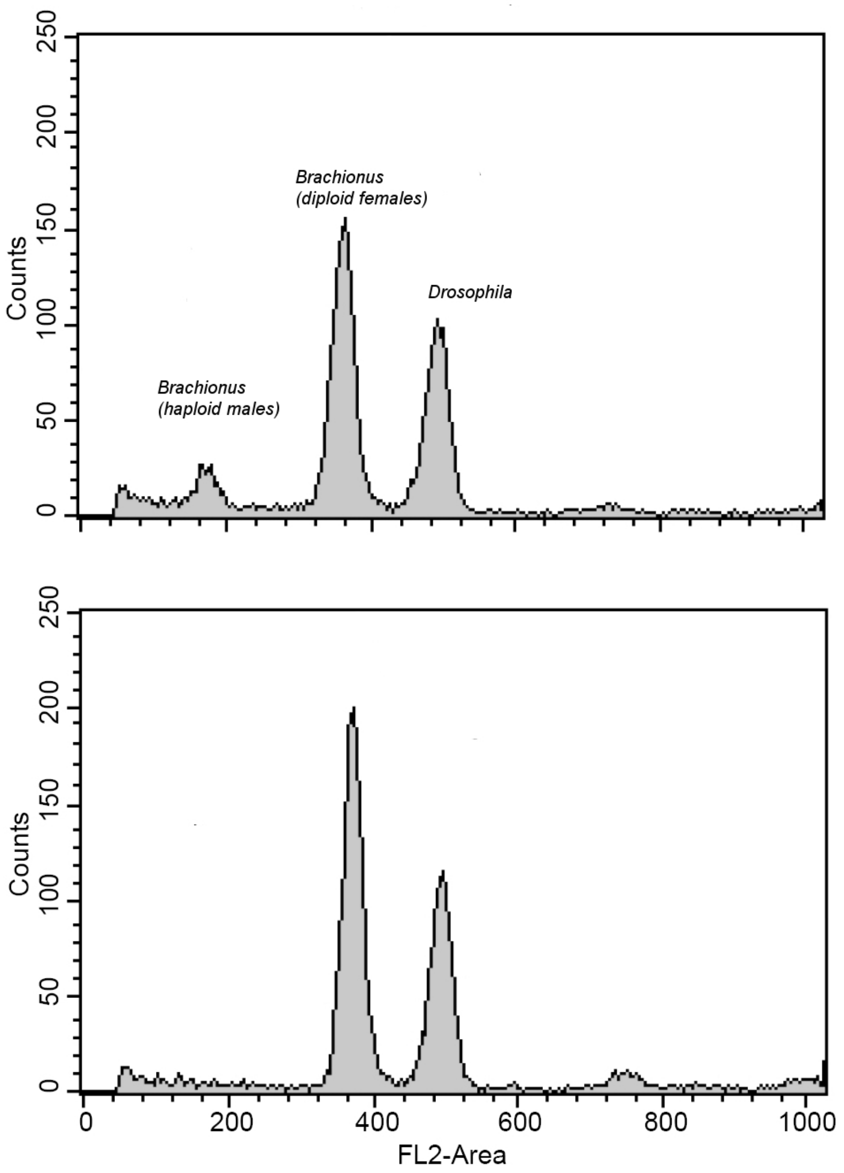

Fig. S3: Example data of flow cytometric measurements. Top: cyclical parthenogenetic clone ("Florida 4"). Bottom: Obligate parthenogenetic clone. Note the small haploid (male) peak in the cyclical parthenogen, having exactly half the fluorescence intensity of the diploid (female) peak.

References

1. Vindeløv LL, Christensen IJ, & Nissen NI (1983) A Detergent-trypsin method for the preparation of nuclei for flow cytometric DNA analysis. *Cytometry* 3(5):323-327.
2. DeSalle R, Gregory TR, & Johnston JS (2005) Preparation of samples for comparative studies of arthropod chromosomes: Visualization, in situ hybridization, and genome size estimation. *Methods in Enzymology* 395:460-488.
